# PVP-coated silver nanoparticles block the transmission of cell-free and cell-associated HIV-1 in human cervical culture

**DOI:** 10.1186/1477-3155-8-15

**Published:** 2010-07-13

**Authors:** Humberto H Lara, Liliana Ixtepan-Turrent, Elsa N Garza-Treviño, Cristina Rodriguez-Padilla

**Affiliations:** 1 Laboratorio de Inmunología y Virología, Departamento de Microbiología e Inmunología, Facultad de Ciencias Biológicas, Universidad Autónoma de Nuevo León, San Nicolas de los Garza, México

## Abstract

**Background:**

Previous *in vitro *studies have demonstrated that polyvinylpyrrolidone coated silver nanoparticles (PVP-coated AgNPs) have antiviral activity against HIV-1 at non-cytotoxic concentrations. These particles also demonstrate broad spectrum virucidal activity by preventing the interaction of HIV-1 gp120 and cellular CD4, thereby inhibiting fusion or entry of the virus into the host cell. In this study, we evaluated the antiviral activity of PVP-coated AgNPs as a potential topical vaginal microbicide to prevent transmission of HIV-1 infection using human cervical culture, an *in vitro *model that simulates *in vivo *conditions.

**Results:**

When formulated into a non-spermicidal gel (Replens) at a concentration of 0.15 mg/mL, PVP-coated AgNPs prevented the transmission of cell-associated HIV-1 and cell-free HIV-1 isolates. Importantly, PVP-coated AgNPs were not toxic to the explant, even when the cervical tissues were exposed continuously to 0.15 mg/mL of PVP-coated AgNPs for 48 h. Only 1 min of PVP-coated AgNPs pretreatment to the explant was required to prevent transmission of HIV-1. Pre-treatment of the cervical explant with 0.15 mg/mL PVP-coated AgNPs for 20 min followed by extensive washing prevented the transmission of HIV-1 in this model for 48 h.

**Conclusions:**

A formulation of PVP-coated AgNPs homogenized in Replens gel acts rapidly to inhibit HIV-1 transmission after 1 min and offers long-lasting protection of the cervical tissue from infection for 48 h, with no evidence of cytotoxicity observed in the explants.

Based on this data, PVP-coated AgNPs are a promising microbicidal candidate for use in topical vaginal/cervical agents to prevent HIV-1 transmission, and further research is warranted.

## Background

Acquired immunodeficiency syndrome (AIDS), the disease caused by human immunodeficiency virus (HIV), is responsible for over two million deaths per year. Highly active anti-retroviral therapy (HAART), a treatment regimen that employs a cocktail of drugs to suppress HIV infection, has significantly improved the quality of life and life expectancy of millions of HIV-infected individuals. Numerous HIV-infected individuals are currently treated with HAART, and these individuals harbor chronic long-term infection; as a result, HIV eventually develops resistance to these drugs, resulting in a need to change medication regimens and a subsequent increase in the cost of treatment [1].

Worldwide, nearly half of all individuals living with HIV are females who have acquired the virus through heterosexual exposure [2]. Although the use of prophylactic agents during sexual intercourse can reduce the transmission of HIV-1, this option is not always feasible for many women due to limited economic options and gender inequality. Women cannot reliably negotiate the use of condoms with their sexual partners [3-5], which leaves them vulnerable to unwanted pregnancy and sexually transmitted infections (STIs), including HIV [6,7].

Consequently, women urgently need infection prevention technology [8] that is within their personal control [9,10]. As the clinical deployment of a safe and effective HIV vaccine is likely to be years away, topical microbicide formulations that are applied vaginally or rectally are receiving increasing attention as an alternative strategy for HIV prevention [11,12].

Infection prevention agents, such as vaginal microbicides, must be controlled by women [13] and provide a defense against HIV infection. As such, a contraceptive microbicide could help prevent unintended pregnancies worldwide. To be a microbicide, these agents must be safe and effective [14] following vaginal or rectal administration [15], should cause minimal or no genital symptoms following long-term repeated usage [16], should act rapidly and should offer long-lasting protection from infection [17].

However, proper evaluation of the efficacy of such agents in blocking HIV infection of female genital tissue has been hampered by the lack of appropriate experimental models [18].

Previously demonstrated with a cervical tissue model, the major target cells of infection reside below the genital epithelium. As a result, HIV must cross this barrier to establish infection. Immune activation due to inflammation secondary to venereal diseases enhances HIV infection of subepithelial cells, suggesting that genital epithelial cells are not susceptible to HIV infection and play no part in the transfer of infectious virus across the epithelium. As a result, these cells may provide a barrier to infection. They also demonstrated that virucidal agents designed for topical vaginal use block HIV infection of genital tissue. Such agents have major implications as microbicides [18].However the application of microbicides directly to the cervical tissue can damage commensal vaginal flora and result in increased inflammation,[19] leaving women susceptible to opportunistic infections and HIV acquisition [20-22]. Therefore, it is necessary that a microbicidal agent possess virucidal, bactericidal, and anti-inflammatory activities. In addition, the treatment of sexually transmitted diseases may decrease the infectivity of HIV-seropositive women by reducing their exposure to HIV-1 in genital secretions [20].

Ideally, a retrovirucidal agent should fulfill several requirements. First, it should act directly on the virus. Dideoxynucleoside antivirals, such as AZT, require cellular metabolic activation and are, therefore, of little use in this respect. Second, a retrovirucide should act at replication steps prior to the integration of proviral DNA into the infected host cell's genome. Although protease inhibitors prevent maturation of newly synthesized viral particles, they are ineffective against pre-existing HIV infection. Third, a retrovirucide should be able to be absorbed by uninfected cells and provide protection from infection by the residual active virus [23,24].

Silver ions have demonstrated activity against both bacteria and viruses. For example, AgNO_3 _has been widely used as a cauterizing agent for patients with aphthous stomatitis [25,26], as a treatment of epistaxis in children [27], and to stanch hemorrhages in cervices following biopsies [28]. Additionally, AgNO_3 _has been used to prevent gonococcal ophthalmia neonatorum in newborns for centuries [29]. Other agents derived from silver, such as silver sulfadiazine (AgSD) cream, have been used by physicians as topical treatments for burn wounds for the past 60 years. During these treatments, erythema decreases, whereas the expression of matrix metalloproteinases (MMPs) increases. This combination reduces chronic inflammation without altering the patients' resistance to bacteria and, importantly, does so without inducing scars.

Recent advances in nanotechnology have resulted in the ability to produce pure silver as nanoparticles [30-35], which are more efficient against HIV than silver ions (AgSD and AgNO_3_) [36]. In addition, silver ions, silver nanoparticles and silver nanocrystals are able to reduce inflammation by altering the levels of cytokines involved in the wound-healing process [37,38]. Decreased levels of IL-10 and IL-6 may be important in preventing the formation of scars during wound repair [37]; as such, silver nanoparticles may represent a possible microbicide alternative for the treatment of HIV-1 [39-42].

According to our previous *in vitro *results, polyvinylpyrrolidone coated silver nanoparticles (PVP-coated AgNPs) inhibit HIV-1 infection (regardless of viral tropism or resistance profile) by binding to gp120 in a manner that prevents CD4-dependent virion binding, fusion, and infection. As such, PVP-coated AgNPs block HIV-1 cell-free and cell-associated infection and act as a virucidal agent. As previously described, PVP-coated AgNPs are an interesting virucidal candidate.

**Figure 1:**
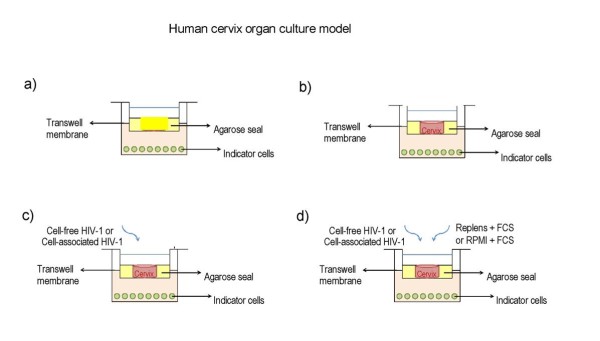
**Human cervical culture model**. **a) **To rule out possible leaks in the agarose seal, Dextran blue was added to the upper chamber on day 6 of culture, and its presence in the lower chamber was determined 20 h later to all Transwells used in the experiments and negative control well with agarose only, b) other negative control with tissue alone without treatment and without challenge with virus and c) positive control well with tissue alone infected with only with HIV-1 virus. d) Inhibition of HIV-1 transmission, Cervical tissue is treated with PVP-coated AgNPs at different concentrations in a Replens gel or RPMI + 10% FCS media, which was then infected with HIV_IIIB_. HIV transmission or inhibition of transmission across the mucosa was determined in the lower chamber by formation of syncytia using indicator cells (MT-2).

Therefore, we investigated the antiviral potency of PVP-coated AgNPs in an *in vitro *human cervical tissue-based organ culture that simulates *in vivo *conditions [36]. We chose this model, as it included all of the natural architecture found *in vivo*: stratified squamous epithelium, submucosa, and immune cells (Fig. 1) [23,24,43]. This model has been used to quantify inhibition of HIV infection transmission throughout a cervical explant in a non cytotoxic range of microbicide. In addition, this model is useful for delimiting the time needed to observe antiviral activity and for defining the duration of protection rendered against infection after application of the gel on human tissue.

## Results

### Toxicity of PVP-coated AgNPs to the cervical tissue

**Figure 2:**
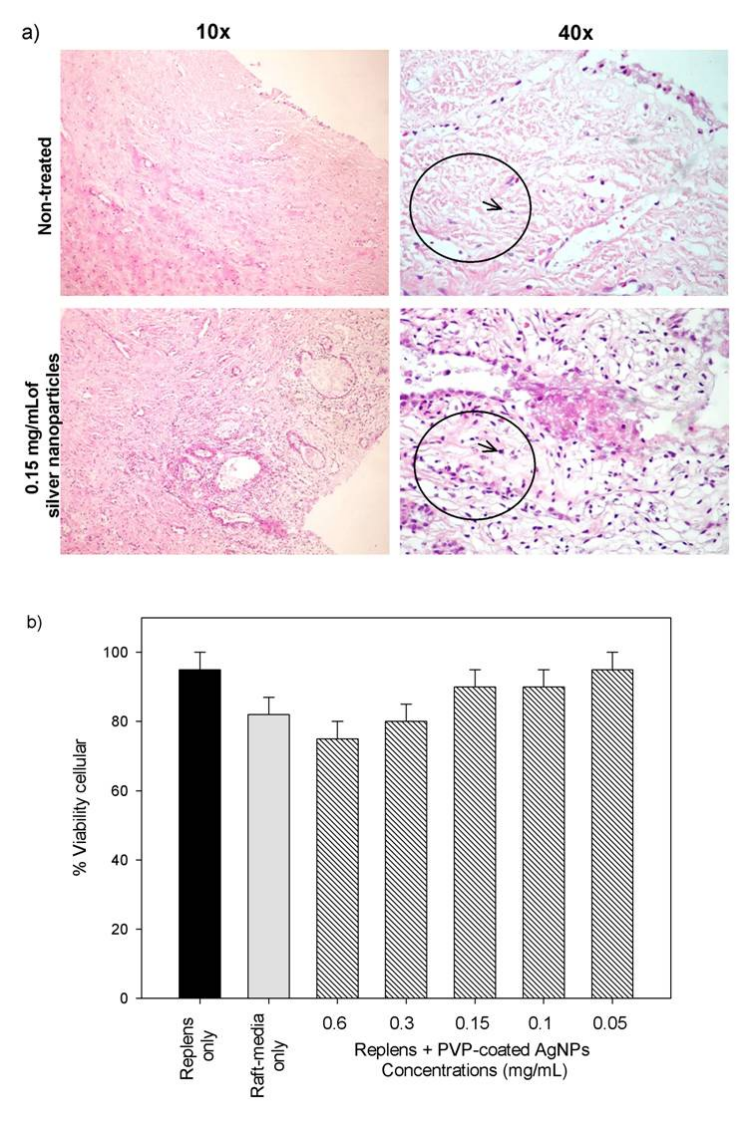
**Toxicity of the PVP-coated AgNPs to the cervical tissue**. a) Normal squamous epithelium and the stroma of ecto-cervical tissues were exposed to Replens gel mixed with 0.15 mg/mL PVP-coated AgNPs for 48 h. Ecto-cervical explants (5 mm) were exposed to either Replens gel alone, which served as a control, or to PVP-coated AgNPs. After 48 h of incubation in a 37°C humidified incubator, the tissues were washed, embedded in paraffin, and stained with hematoxylin and eosin. b) Cervical explants in the upper Transwell chambers were exposed to Replens alone as a control, Raft-medium, or Replens gel containing different concentrations of PVP-coated AgNPs (0.6, 0.3, 0.15, 0.1, 0.05 and 0 mg/mL). After 24 h, the medium containing PVP-coated AgNPs was removed and washed three times with culture media. Cell viability was measured by the CellTiter-Glo^® ^assay. Graphs show values of the means ± standard deviations from three separate experiments. Graphs were created using the SigmaPlot 10.0 software.

To determine the toxic effect of PVP-coated AgNPs, we analyzed the cervical stroma using hematoxylin and eosin staining. First, we treated ecto-cervical tissues with 0.6, 0.3, 0.15, 0.1 and 0.05 mg/mL PVP-coated AgNPs for 48 h. The highest dose of PVP-coated AgNPs (0.6 mg/mL) did not cause an acute inflammatory response in the cervical explant or induce cell death, as determined by the histopathology with no signs of cell damage (no edema, eosinophils or apoptosis). Compared to the negative control (no treatment), cervical tissue that had been incubated with 0.6, 0.3, 0.15, 0.1, and 0.05 mg/mL PVP-coated AgNPs for 48 h showed mild lymphoid infiltration compared with the negative control (Fig 2a).

Next, we evaluated cell viability after 24 h of treatment with various concentrations of PVP-coated AgNPs (0.6, 0.3, 0.15, 0.1 and 0.05 mg/mL) by comparing the percentage of viable cells in the cervical tissue without treatment with PVP-coated AgNPs, relative to the positive control, which was measured as the amount of ATP released from viable cells using a luciferase-based assay. The values of PVP-coated AgNPs chosen for toxicity studies exceeded the amount necessary to inhibit transmission of HIV-1 infection *in vitro *trough the cervical explant [36]. Treatment with 0.3 mg/mL PVP-coated AgNPs was cytotoxic in only 20% of the cells of the cervical explant, whereas a dose of 0.6 mg/mL was cytotoxic to 23% of the cells. PVP-coated AgNPs formulated in Replens gel inhibited cell viability by 5%, and Raft-media was cytotoxic to 18% of cells (Fig. 2b). Raft-media has many antibiotics, which, when combined, result in cytotoxicity.

### Inhibition of cell-free and cell-associated HIV-1 viral infection in the presence or absence of Replens gel

**Figure 3:**
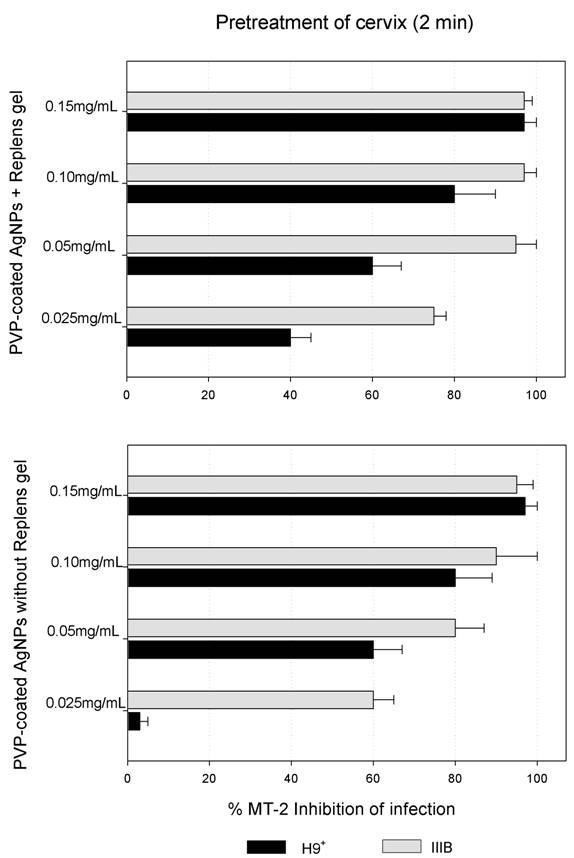
**Inhibition of HIV-1 transmission with and without Replens gel using the cervical culture model**. The upper chamber of the Transwell with the cervical explant was exposed for 2 min to different concentrations of PVP-coated AgNPs (0.025, 0.05, 0.1 and 0.15 mg/mL), either alone or mixed with the Replens gel. After thoroughly washing extracellular PVP-coated AgNPs from the cervical explant, cell-free (HIV-1_IIIB_) [(5 × 10^5 ^TCID_50_)], or cell-associated virus (H9^+^) (5 × 10^5 ^cells) were added. To evaluate inhibition of the HIV-1 infection, indicator cells (MT-2) in the lower chamber were cultured and formation of syncytia was monitored for ten days. Graphs show values of the means ± standard deviations from three separate experiments. Graphs were created using the SigmaPlot 10.0 software.

Results showed that two minutes of pre-treatment of the cervical explant with 0.025 to 0.15 mg/mL of PVP-coated AgNPs formulated in the Replens gel protected the cervical tissues from HIV-1_IIIB _infection; this inhibitory effect was independent of the effect of the Replens gel alone. In addition, PVP-coated AgNPs with or without the Replens gel completely neutralized cell-free and cell-associated HIV-1 transmission of infection through cervical tissues at a dose of 0.15 mg/mL, although a similar result was obtained at doses of 0.1 and 0.05 mg/mL Replens gel conferred protection in a dose-dependent manner, inhibiting infection associated with the (H9+) cells more efficiently than PVP-coated AgNPs in RPMI media containing 10% FCS alone; the result was most significant at a dose of 0.025 mg/mL (Fig. 3).

### Minimal time of exposure to PVP-coated AgNPs needed to confer protection against the HIV-1 transmission of cell-associated infection in the cervical culture model

**Figure 4:**
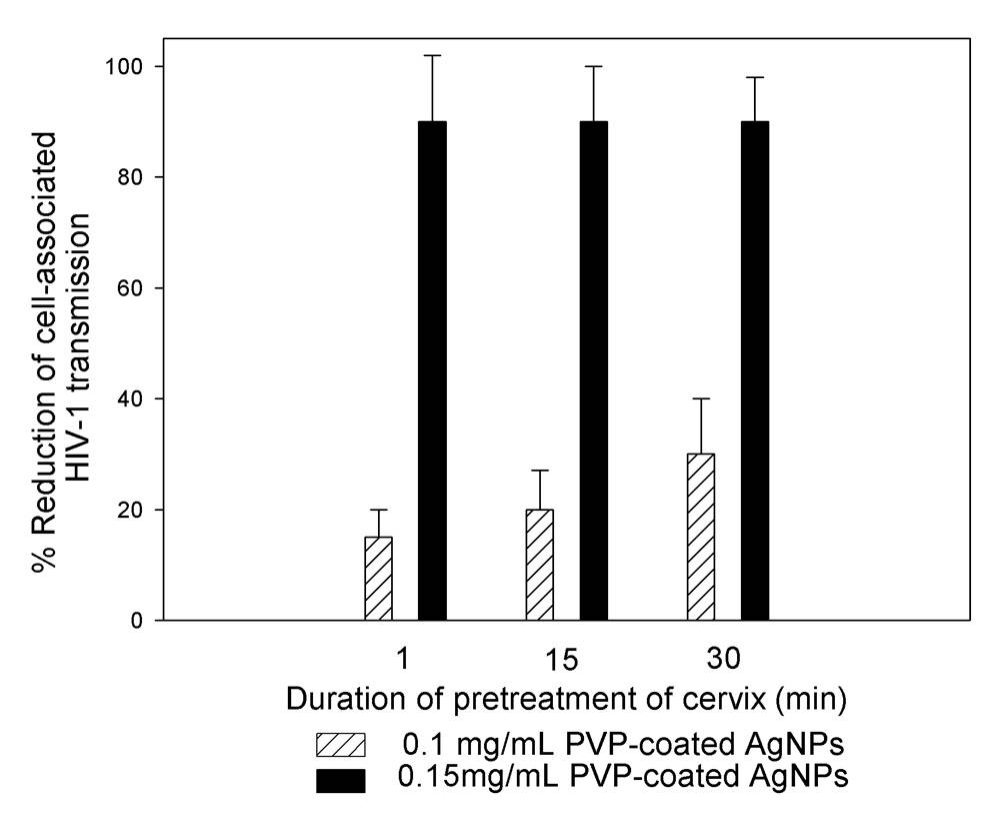
**Time needed for PVP-coated AgNPs to confer protection from the transmission of HIV-1 through the cervical**. Cervical explants were pretreated for 1, 15 and 30 minutes with 0.1 or 0.15 mg/mL PVP-coated AgNPs. After thoroughly washing extracellular PVP-coated AgNPs from the cervical tissue, cell-associated virus (H9^+^) (5 × 10^5 ^cells) was added to the upper chamber of the Transwell. Indicator cells (MT-2) were cultured in the lower chamber to evaluate the inhibition of HIV_IIIB _infection. Graphs show values of the means ± standard deviations from three separate experiments. Graphs were created using the SigmaPlot 10.0 software.

We evaluated the time required for 0.1 and 0.15 mg/mL doses of PVP-coated AgNPs to block HIV-1_IIIB _infection of cell-associated (H9 +) transmission through the cervical tissue. Complete protection occurred within one minute of pre-treatment with 0.15 mg/mL PVP-coated AgNPs incubated for different times and after washing away of the extracellular drug. Furthermore, PVP-coated AgNPs completely blocked the T tropic wild type (HIV-1_IIIB_) virus, the drug resistant viral isolate (AZT-_RV_), and cell-associated HIV-1 (H9^+ ^cells) transmission through cervical tissue after one minute of pre-treatment (Fig. 4).

### Duration of the protection time from HIV-1 infection following 20 minutes pre-treatment of the cervical explants with PVP-coated AgNPs

**Figure 5:**
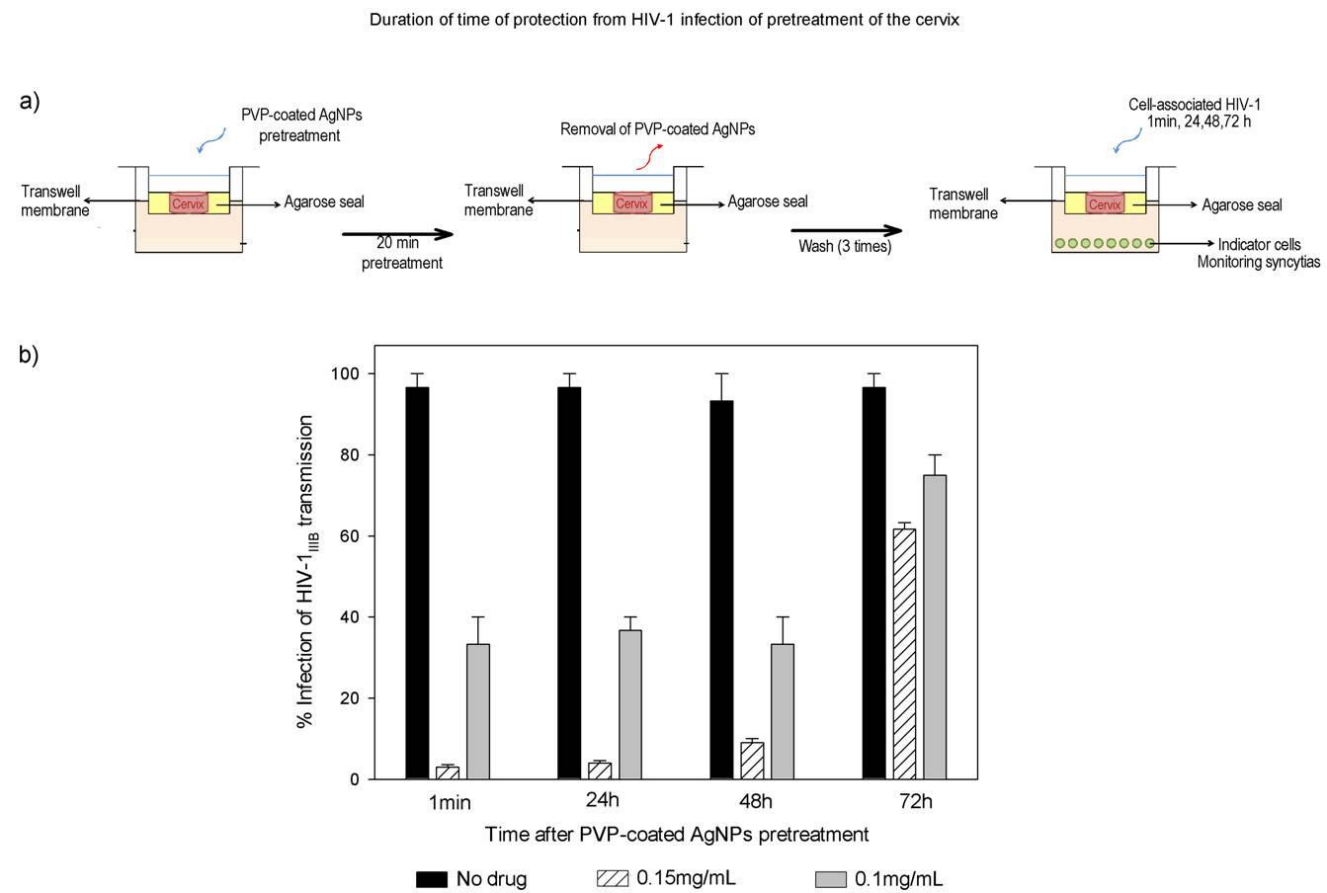
**Protection from HIV-1 infection following pre-treatment of the cervical explant with PVP-coated AgNPs**. **a) **Cervical explants were exposed to 0.1 or 0.15 mg/mL PVP-coated AgNPs in RPMI + 10% FCS media for 20 minutes. After thoroughly washing extracellular PVP-coated AgNPs from the cervical explant and after 1 minute, 24 h, 48 h and 72 h, cell-free virus (HIV-1_IIIB_) [(5 × 10^5 ^TCID_50_)] was added to the upper chamber. To verify the neutralization of HIV-1 transmission, we cultured the indicator cells (MT-2) in the lower chamber and evaluated inhibition of the HIV-1 infection. b) Cervical explants were exposed to HIV-1 in the absence of PVP-coated AgNPs as a control and to 0.1 or 0.15 mg/mL of PVP-coated AgNPs as pretreatment. Graphs show values of the means ± standard deviations from three separate experiments. Graphs were created using the SigmaPlot 10.0 software.

After 20 minutes of pre-treatment of the cervical explants with 0.15 mg/mL PVP-coated AgNPs, the drug was removed and washed from the upper chamber, which conferred almost total protection (90%) against HIV-1 transmission of infection for 48 h (Fig. 5), indicating a long-lasting protective effect by the PVP-coated AgNPs in the cervical explant.

## Discussion

The development of non-toxic microbicides effective against the transmission of cell-free and cell-associated virus, which have long-lasting efficacy on the treated tissue [17,44] and are rapidly acting [45], is a highly desirable approach to the prevention of HIV-1 transmission during sexual intercourse. Inhibiting the transmission of HIV *in vivo *will likely require a combination of microbicidal products that provide broad anti-viral effects and prevent the development of HIV strains resistant to the microbicides [46]. It is clear that the development of a topical vaginal microbicide is technically, ethically, and culturally complicated. However, the number of lives saved with such an agent may exceed the risks involved [47].

In fact, microbicides could have the potential to eliminate drug-resistant bacteria, in addition to sexually transmitted diseases that cause inflammation. Previous studies have reported that silver ions and silver nanoparticles exert anti-inflammatory effects, induce lymphoproliferation, and inhibit bacterial and HIV-1 infection. These characteristics make silver nanoparticles of interest in microbicide research [48,49].

The mechanism of the antiviral action of PVP-coated AgNPs as an HIV-1 virucidal agent has been previously established by Lara HH et al. First, studies have revealed that PVP-coated AgNPs inactivate HIV-1 and block viral entry through gp120-CD4 interaction. Second, PVP-coated AgNPs (1.0-2.5 mg/mL) efficiently block the fusion of HL2/3 and HeLa CD4 cells in a dose-dependent manner. Third, PVP-coated AgNPs act as an effective broad-spectrum microbicide against cell-free virus (i.e., laboratory strains, clinical isolates, T- and M-tropic strains, and resistant strains), as well as the cell-associated virus. Fourth, PVP-coated AgNPs are effective virucides, as they inactivate HIV particles in a short period of time, exerting their activity at an early stage of viral replication (i.e., entry or fusion) and at post-entry stages [36].

Recent studies have shown that silver nanoparticles are capable of being internalized into cells and can penetrate through skin cells (HEKs) [50]. Other authors have described the localization of PVP-coated AgNPs only in the superficial layers of the stratum corneum, a result similar to that found in a static cell diffusion study [51]. Other nanoparticles have not been shown to penetrate into the deeper epidermis [52,53].

Finally, previous studies report that silver nanoparticles and silver nanocrystals suppress the expression of TNF-α, which is a cytokine that plays a pivotal role in HIV-1 pathogenesis by up-regulating the transcription of HIV-1 [48,49]. It also prevents inflammation and, as such, may enhance wound healing *in vivo *[54]. Moreover, inflammation produces immune activation, enhancing HIV-1 infection of subepithelial cells of the human cervical tissue. Consequently, an agent that prevents inflammation should inhibit the transmission of HIV infection by impeding the enhancement of HIV infection [18].

Based on the previous studies mentioned above, the purpose of this study was to demonstrate the ability of PVP-coated AgNPs to inhibit HIV-1 transmission of infection in a rapid manner with long-lasting effects and efficiently throughout the human cervical explant in an *in vitro *model that simulates *in vivo *conditions.

For these studies, we used a model of a human cervical explant, which contained the natural *in vivo *tissue architecture of stratified squamous epithelium, submucosa, and immune cells (Fig. 1). In this model, the infectious virus is transmitted across the mucosal barrier by both cell-free and cell-associated HIV-1 [7]. Although some researchers have questioned whether HIV transmission in this model is a result of leakage around the polarized cervical tissues [55], these concerns were rebutted [56] based on additional data not shown in the original description of the model. This model has also been used by Greenhead and colleagues to study the effects of various microbicides [57].

PVP-coated AgNPs that were formulated using Replens gel were more effective as a virucide compared to the PVP-coated AgNPs dissolved in RPMI+10% FCS media. This increased activity is due to the ability of this gel to diffuse the PVP-coated AgNPs more homogenously into the cervical tissue as compared to the medium [6] (Fig. 3), even though RPMI with FCS prevents agglomeration of PVP-coated AgNPs [58]

We demonstrated that pre-treatment of cervical tissues with PVP-coated AgNPs neutralized the transmission of HIV-1 using human cervical explants. Specifically, we found that 0.15 mg/mL PVP-coated AgNPs inhibited infection by HIV-_IIIB _and HIV-_AZT-RV _cell-free viruses as well as cell-associated infection at doses that were not toxic to the human cervical tissue. In addition, treatment of the cervical tissue with 0.15 mg/mL PVP-coated AgNPs augmented the number of lymphocytes relative to the control (Fig 2a). The increased proliferation of lymphocytes was presumably due to activation of the immune system, which was induced by the continuous expression of death factors (mutations of Fas-L or CD95). This resulted in activation of lymphocytes, including CD4 T cells, CD8 CTL, or APC and turned them into effectors of apoptosis, leading to the destruction of healthy, non-infected cells [59-61]. Silver ions and silver nanoparticles improve wound healing by reducing inflammation, inducing the proliferation of lymphocytes [37] and inhibiting bacterial and HIV-1 infection; thus, PVP-coated AgNPs are a potential therapeutic agent against the dissemination of drug-resistant bacteria, thereby providing protection from sexually transmitted diseases. Importantly, we demonstrated that high concentrations of PVP-coated AgNPs (0.3 and 0.6 mg/mL) were only cytotoxic to a small population of cells, affecting the viability of 20-23% of the cells in the cervical explant, which correlates with low off-target cytotoxic effects (Fig. 2).

An ideal microbicide should act rapidly [45]; in accordance with this, we observed that one minute of exposure to PVP-coated AgNPs (0.15 mg/mL) was the minimal time necessary to achieve protection of the cervical tissue against the transmission of infection by cell-free and cell-associated viruses (Fig. 4). Previously, *in vitro *studies demonstrated that when PVP-coated AgNPs (2.5-5 mg/mL) were dissolved in RPMI+10% FCS media, they conferred partial protection (50%) from HIV-1 cell-associated infection in a dose-dependent manner [36]. In further support of PVP-coated AgNPs as microbicides, PVP-coated AgNPs were also effective in the presence of cell-associated infection, even under 'non-optimal' conditions.

First-generation microbicides are only effective for a few hours and, therefore, require administration shortly before coitus [62]. Previously, we reported that treatment with PVP-coated AgNPs in concentrations ranging from 0.031 mg/mL to 5.0 mg/mL for one minute reduced the transmission of HIV-1 infection in PBMC and H9+ cells by 20%-30% and that this protection lasted for several hours [36].

Using the cervical explant model, we evaluated the long-term effectiveness of PVP-coated AgNPs, which is an important pharmacodynamic parameter investigated during the development of a topical microbicide agent. It contributes to the choice of antiviral dosing regimens and is defined as the length of time that infection is suppressed following brief exposure to the antimicrobial agent. Ideally, a microbicide should remain effective for several hours after topical application [63]. In this study, the transmission of HIV-1 infectivity through the cervical explant was inhibited in almost all cases when PVP-coated AgNPs were formulated in the Replens gel. Additionally, PVP-coated AgNPs (0.15 mg/mL) that were applied to the cervical tissue for 20 min in a gel formulation were able to abolish HIV-1 transmission for a period of 48 h after the gel was removed and washed thoroughly; after this period of time, the HIV-1 was added to the cervical explant at different times until 72 hours to evaluate the duration of protection to the tissue (Fig. 5). These results are in accordance with our previous findings that showed pre-treatment of uninfected cells with PVP-coated AgNPs conferred protection from acquiring HIV-1 *in vitro*, even in the absence of extracellular drug [36]. A dose of 0.15 mg/mL PVP-coated AgNPs represents a threshold level necessary for inhibition of transmission, even after 48 h (Fig 5). PVP-coated AgNPs were able to confer protection for similar lengths of time compared to other microbicides, including UC781 [64,23,24,66].

In comparison to various viral entry inhibitors, PVP-coated AgNPs offer many advantages. For example, although dextrin sulfate reduced the ability of virus (HIV-1HSBc2) to infect cells *in vitro *by 77%, it did not protect cells against the R5 virus (HIV-1 JRCSF) [67]. Further, although nonoxynol-9 is a microbicide that is active against a wide range of pathogens, it is potentially cytotoxic to host cells. In contrast to these compounds, PVP-coated AgNPs have low cytotoxicity, protect cervical tissue against HIV infection in a manner independent of co-receptors [36] and could possibly reduce inflammation. As such, PVP-coated AgNPs are an ideal microbicide to study [68].

Our hypotheses concerning the inactivation of HIV-1 transmission throughout the cervical explant model using the PVP-coated AgNPs is that the drug acts as a potent virucidal agent that attaches at the viral membrane, [36,69] and may protect the natural barrier of the genital epithelium, therefore inactivating the ability of the HIV virus to reach the target cells that reside below. Thus, when the HIV virus crosses the genital epithelium, it is already inactivated and unable to transfer infection to the target cells that reside in the subepithelium [36], as evidenced by an absence of infection (absence of syncytia on indicator cells) on the lower chamber of the cervical model after treatment with PVP-coated AgNPs.

In addition to their virucidal activity, PVP-coated AgNPs also impair the ability of the HIV-1 virus to develop resistance [36]. Importantly, these nanoparticles have potent activity against most strains of HIV and provide broad protection against other STIs. These compounds are stable at room temperature, accessible in terms of cost, and have demonstrated *in vitro *safety [36,70,71].

## Conclusions

Previous *in vitro *studies evaluating PVP-coated AgNPs as potential virucidal agents have revealed that these compounds, in addition to providing broad-spectrum bactericidal and HIV-1 virucidal activity, also blocked the infection of cell-free and cell-associated HIV-1 [72]. The gel formulation of PVP-coated AgNPs is probably the first microbicide with broad spectrum virucidal, bactericidal, and anti-inflammatory properties in cervical tissue [36,54,73].

Our results show that PVP-coated AgNPs function as potential microbicides with virucidal properties that are capable of preventing the transmission of HIV-1 in a human cervical tissue explant model when used at a nontoxic dosage range. PVP-coated AgNPs protect against infection transmission of cell-free and cell-associated HIV-1, acting within one minute after the treatment of the cervical tissue. Importantly, after 20 minutes of pre-treatment with PVP-coated AgNPs and subsequent washing, the cervical culture remained protected against infection with HIV-1 for as long as 48 h, demonstrating long-lasting protection. This feature is necessary for a topical vaginal microbicide to ensure protection many hours after gel application and even more so after the gel is washed away [17].

However, further studies are necessary to evaluate the potential toxicities (i.e., genetic, reproductive, and carcinogenic toxicities) and long-term side effects associated with the use of PVP-coated AgNPs as an inhibitor of HIV-1 infection. Studies evaluating hypersensitivity/photosensitivity and condom integrity are also necessary [7].

## Methods

### Silver Compounds

Commercially manufactured 30-50 nm spherical silver nanoparticles surface-coated with 0.2 wt% PVP (PVP-coated AgNPs) were used for these studies (NanoAmor, Houston, TX). Stock solutions of PVP-coated AgNPs were prepared in RPMI 1640 cell culture media with 10% FCS.

Serial dilutions of the stock solution were made using RPMI + 10% FCS media.

### HIV-1 isolates and cell culture

The following reagents were obtained from the AIDS Research and Reference Reagent Program, AIDS Division, the National Institute of Allergies and Infectious Diseases and the National Institute of Health and Collaborators: MT-2 (from Dr. Douglas Richman), H9^+ ^cells (from Dr. Robert Gallo), HeLa-CD4-LTR-β-gal cells and the viral strains HIV-1_IIIB _and HIV-1_AZT-RV_. HIV-1_IIIB _was propagated by sub-culturing in the MT-2 and H9^+ ^cells, according to the DAIDS Virology Manual for HIV Laboratories. Aliquots of the cell-free supernatants from virulent cultures were used for viral inoculation. MT-2 and H9^+ ^cells were cultured in RPMI 1640 (Sigma-Aldrich), supplemented with 10% fetal calf serum (FCS) and antibiotics. Commercially manufactured 30-50 nm PVP-coated AgNPs were used in these studies (NanoAmor, Houston, TX). A stock solution was prepared in RPMI culture media enriched with 10% fetal calf serum (FCS), which prevents agglomeration. Serial dilutions of the stock solution yielded four different solutions with concentrations ranging from 0.025 to 0.6 mg/mL All work related to HIV-1 and cell culture manipulation was done in a biosafety level 3 (BSL-3) laboratory at the Laboratorio de Inmunología y Virología, Universidad Autonoma de Nuevo Leon, Mexico.

### Formulation of Replens gel/PVP-coated AgNPs

PVP-coated AgNPs were formulated in a non-spermicidal Replens gel (3% glycerin, 0.08% sorbic acid, 1% carbopol 940, 4% liquid paraffin and 16% 1 N NaOH) [24]. Gels containing PVP-coated AgNPs at serial concentrations from 0.025- 0.6 mg/mL were added to the upper chambers of the cervical culture model.

### Toxicity of PVP-coated AgNPs to the cervical tissue

The effect of various concentrations of PVP-coated AgNPs (0.6, 0.3, 0.15, 0.1 and 0.05 mg/mL) on cervical explant tissues for 48 h was examined by histochemistry, as previously described [23,24]. The viability of cervical biopsies was quantified after 24 h of exposure to Replens gel mixed with 0.6, 0.3, 0.15, 0.1 and 0.05 mg/mL PVP-coated AgNPs, using the CellTiter-Glo^® ^luminescent cell viability assay (Promega Cat. G7572). Microtiter plates were incubated at 37°C in a 5% CO_2 _humidified atmosphere for 24 h and were used to determine the number of viable cells in a culture by quantification of ATP. All assays were run in parallel according to the producer's protocol and included both a negative (measure of only reagent) and positive control (explant without treatment). A Veritas microplate luminometer from Turner Biosystems (Model 9100-002) was used in these experiments. Cytotoxicity was evaluated in a dose-dependent manner and was based on the percentage of viable cells relative to the positive control.

### Cervical explant model

Ecto-cervical tissue was collected from HIV-1-negative, pre-menopausal women undergoing planned therapeutic hysterectomies after their informed consent was obtained. All tissues were processed for organ culture within 5 hours of the completion of surgery. Tissue samples were soaked in a concentrated antibiotic wash solution (20,000 U/mL penicillin and streptomycin, 250 μg/mL fungizone, and 120 U/mL nystatin) for 10 min. The tissues were then washed three times in Raft-media 21 (Dulbecco's modified Eagle medium supplemented with 25% Ham's F12 medium, 0.1 nM cholera toxin, 5 μg/mL apo-transferrin, 4 mg/m/L hydrocortisone, 0.5 ng/mL EGF, 10% FBS and 10,000 U/mL penicillin, and streptomycin) and cut into 0.4 × 0.5 cm pieces. A piece of tissue with the epithelial layer oriented on top was placed in the top chamber of a 12-well Transwell plate, a permeable tissue culture support that uses microporous membranes. These permeable wells permit cells to uptake and secrete molecules on both their basal and apical surfaces and, thereby, carry out metabolic activities in a more physiological fashion. A 3% solution of agarose in Hank's medium was added to the area surrounding the tissue in the top well, which upon solidification created a tight seal around the tissue. Cervical and vaginal explants, comprising epithelial and stromal tissues, were kept at 37°C in a humidified atmosphere containing 5% CO_2 _[23,74].

To rule out possible leaks in the agarose seal, Dextran blue was added to the upper chamber on day 6 of culture, and its presence in the lower chamber was determined 20 h later[13].

### Inhibition of cell-free and cell-associated HIV-1 viral infection in the presence or absence of Replens gel

In these experiments we used an *in vitro *cervical tissue-based organ culture model that was developed to study the heterosexual transmission of HIV-1 infection simulating *in vivo *conditions.

The upper chamber of the Transwell with cervical explant was pre-treated for 2 min at different concentrations of PVP-coated AgNPs (0.025, 0.05, 0.1 and 0.15 mg/mL), either alone or in formulation with the Replens gel, followed by thorough washing of the extracellular PVP-coated AgNPs from the cervical explant. Cell-free (HIV-1_IIIB_) [(5 × 10^5 ^TCID_50_)] or cell-associated virus (H9^+^) (5 × 10^5 ^cells) was added to the cervical explant in the upper chamber. To evaluate the inhibition of HIV-1 transmission through the cervical explant, indicator cells (MT-2) were cultured in the lower chamber and were monitored for the formation of syncytia for ten days, as previously described [13,75,76]. A positive virus control (cervical explant infected with HIV-1 without treatment) must produce observable syncytia within seven days of incubation, which reflects the presence of infection. The first reading of the plate must be made by day three. Negative control wells (cervical explants not infected with HIV-1) must not develop syncytia, which reflect an absence of infection. If either control does not react as expected, the assay is suspect and should be repeated.

In the case of the MT-2 cells, half of the media were changed for new RPMI+10% FCS media with MT-2 uninfected cells every three days, and the formation of syncytia was monitored for ten days. The cytopathic effects of the viral infection of MT2 cells were analyzed by microscopic assessment of syncytia formation. These latter data were obtained by analysis of duplicate samples by two independent observers [13,75,76].

### Minimal time of exposure to PVP-coated AgNPs needed to confer protection from HIV-1 transmission of cell-associated infection in the cervical culture model

To define the minimal time of exposure needed to confer protection to the cervical explant from transmission of infection, RPMI+10% FCS media containing 0, 0.1 and 0.15 mg/mL PVP-coated AgNPs was added to the upper chambers, and 1, 15, or 30 minutes later, the medium was removed. The upper chambers were then washed three times with the culture media. H9^+ ^(5 × 10^5 ^cells) were then added to the upper chamber of the Transwell to evaluate inhibition of HIV-1 cell-associated transmission. Target cells (MT- 2) were added to the lower chambers. In the case of the MT-2 cells, half of the media were replenished with the new media and added to the MT-2 uninfected cells every three days. The formation of syncytia was monitored for ten days.

### Duration of time of protection from HIV-1 infection following 20 minutes of pre-treatment of the cervical explants with PVP-coated AgNPs

Five millimeter circular pieces of ectocervical tissues were placed in the top chambers of a 12-well Transwell sealed with 3% agarose with the epithelial layer oriented on top. Media containing PVP-coated AgNPs was added to the upper chambers, and after 20 min of treatment, the RPMI+10% FCS media containing PVP-coated AgNPs in the upper chambers was removed from ectocervical tissues on the upper chambers and was then washed thoroughly three times with media. After washing of the top chambers, HIV-1_IIIB _[(5 × 10^5 ^TCID_50_)] was added after 1 min, 24 h, 48 h and 72 h. Indicator cells (MT- 2) were added to the lower chambers to measure the percentage of inhibition of infection transmission through the cervical explant to the lower chamber where the indicator cells are cultured. With respect to the culture of MT-2 cells, half of the media were exchanged for new media and added to the MT-2 uninfected cells every three days. The formation of syncytia was monitored for ten days after infection.

### MT-2 infectivity assay

MT-2 cells were added as indicator cells to monitor the transmission of HIV-1 infectivity to the lower chambers as soon as the HIV-1 was added to infect the cervical tissue of the upper chamber with or without PVP-coated AgNPs formulated into gel or RPMI+10% FCS media. In the case of MT-2 cells, half of the media was replenished with the new media and added to the MT-2 uninfected cells every three days. The formation of syncytia was monitored every day for ten days. For a positive control on cervical tissue, only HIV_IIIB _was added without treatment with PVP-coated AgNPs, syncytia were counted for all cells in the tissue. For the negative control, only cervical tissue without HIV_IIIB _and PVP-coated AgNPs were used. Syncytia were counted in the lower chamber; for the negative control, all cells were expected to be without syncytia.

The percentages of cells with tissue showing signs of inhibition of HIV-1 infection transmission were evaluated with respect to the positive control. The cytopathic effects of the viral infection of MT2 cells were analyzed by microscopic assessment of syncytial formation. These latter data were obtained by analysis of duplicate samples by two independent observers [75-77].

### Statistical analysis

Graphs show values of the means ± standard deviations from three separate experiments. Graphs were created using the SigmaPlot 10.0 software.

## Competing interests

The authors declare that they have no competing interests.

## Authors' contributions

All authors read and approved the final manuscript. H.H.L. participated in the conception and experimental design of the *in vitro *HIV-1 manipulation and infection assays, in the analysis and interpretation of the data, and in the writing and revision of this report. L.I-T. participated in designing the *in vivo *cervical tissue model and helped analyze and interpret the results. H.H.L. and L.I-T. made equal contributions to this study working in the cervical model, working designing, and authoring E.N.G-T. participated in the analysis and interpretation of the data and in writing and revising this report. C.R-P. participated in the experimental design of this study.
